# Effects of peroxisome proliferator activated receptors (PPAR)-γ and -α agonists on cochlear protection from oxidative stress

**DOI:** 10.1371/journal.pone.0188596

**Published:** 2017-11-28

**Authors:** Marijana Sekulic-Jablanovic, Vesna Petkovic, Matthew B. Wright, Krystsina Kucharava, Nathan Huerzeler, Soledad Levano, Yves Brand, Katharina Leitmeyer, Andrea Glutz, Alexander Bausch, Daniel Bodmer

**Affiliations:** 1 Department of Biomedicine, University Hospital Basel, University of Basel, Basel, Switzerland; 2 Clinic for Otolaryngology, Head and Neck Surgery, University Hospital Basel, Basel, Switzerland; 3 Strekin AG, Basel, Switzerland; Universite Clermont Auvergne, FRANCE

## Abstract

Various insults cause ototoxicity in mammals by increasing oxidative stress leading to apoptosis of auditory hair cells (HCs). The thiazolidinediones (TZDs; e.g., pioglitazone) and fibrate (e.g., fenofibrate) drugs are used for the treatment of diabetes and dyslipidemia. These agents target the peroxisome proliferator-activated receptors, PPARγ and PPARα, which are transcription factors that influence glucose and lipid metabolism, inflammation, and organ protection. In this study, we explored the effects of pioglitazone and other PPAR agonists to prevent gentamicin-induced oxidative stress and apoptosis in mouse organ of Corti (OC) explants. Western blots showed high levels of PPARγ and PPARα proteins in mouse OC lysates. Immunofluorescence assays indicated that PPARγ and PPARα proteins are present in auditory HCs and other cell types in the mouse cochlea. Gentamicin treatment induced production of reactive oxygen species (ROS), lipid peroxidation, caspase activation, PARP-1 cleavage, and HC apoptosis in cultured OCs. Pioglitazone mediated its anti-apoptotic effects by opposing the increase in ROS induced by gentamicin, which inhibited the subsequent formation of 4-hydroxy-2-nonenal (4-HNE) and activation of pro-apoptotic mediators. Pioglitazone mediated its effects by upregulating genes that control ROS production and detoxification pathways leading to restoration of the reduced:oxidized glutathione ratio. Structurally diverse PPAR agonists were protective of HCs. Pioglitazone (PPARγ-specific), tesaglitazar (PPARγ/α-specific), and fenofibric acid (PPARα-specific) all provided >90% protection from gentamicin toxicity by regulation of overlapping subsets of genes controlling ROS detoxification. This study revealed that PPARs play important roles in the cochlea, and that PPAR-targeting drugs possess therapeutic potential as treatment for hearing loss.

## Introduction

Sensorineural hearing loss occurs as a consequence of degeneration and apoptosis of auditory hair cells (HCs) and spiral ganglion neurons (SGNs). Sensorineural hearing loss is a global health problem with profound socioeconomic impact and high unmet medical need. There are currently no satisfactory effective medical treatments for preventing sensorineural hearing loss. Currently, the only treatment options available are offered by devices such as hearing aids and cochlear implants [1].

It has become recognized that the formation of oxygen-free radicals is a key mediator in several types of hearing loss [2, 3]. Reactive oxygen species (ROS) that have been identified in cochlear tissue are derived from mitochondrial production and include hydroxyl radicals, superoxide anions, and hydrogen peroxide [4, 5]. Accumulation of ROS overwhelms endogenous detoxification pathways leading to oxidative modification and damage to lipids, proteins, and nucleic acids. Ultimately, pro-apoptotic signaling pathways, including the c-Jun-N-terminal kinase and P38 MAPK pathways are activated, which may induce HCs to undergo apoptotic cell death [6]. Oxidative stress, with consequent tissue injury, occurs due to the imbalance between the production and removal of ROS. Consistent with this notion, several antioxidants and agents that enhance intrinsic antioxidant defenses have been shown to protect cochlear HCs from apoptosis in the presence of various damage-inducing factors [7].

Peroxisome proliferator-activated receptors (PPARs) are members of the family of ligand-regulated nuclear hormone receptors. In response to various ligands, these transcription factors (PPARα, β/δ and γ) regulate the expression of genes involved in a variety of physiological processes, including lipid and glucose homeostasis, inflammation, and organ protection [8]. PPARγ activity can be stimulated by members of the thiazolidinedione (TZDs) class of anti-diabetic drugs including pioglitazone and rosiglitazone. The TZDs are effective treatments for type 2 diabetes by improving tissue insulin sensitivity and glucose homeostasis. The TZDs have demonstrated diverse pleiotrophic effects in many tissues where they exhibit anti-inflammatory, anti-proliferative and tissue protective effects. PPARα activity can be stimulated by the fibrate drugs such as fenofibrate and gemfibrozil. Fibrates have been employed for many decades for the treatment of dyslipidemia and to reduce cardiovascular risk [8–10].

The present study aimed to investigate the protective capacity of pioglitazone against gentamicin-induced ototoxicity and to extend these observations to other PPAR agonists. We investigated whether the known effects of pioglitazone on inflammation, oxidative stress, and organ protection might extend to the protection of auditory HCs and prevention of hearing loss. We demonstrated that PPARγ and PPARα are highly expressed in several cell types of mouse cochlea, including inner and outer HCs. We found that, in isolated cochlear sensory epithelium, pioglitazone as well as structurally unrelated PPAR agonists with diverse receptor binding selectivity and potency, consistently protected HCs from gentamicin-induced toxicity. Indeed, pioglitazone treatment prevented the increase in ROS induced by gentamicin, inhibited subsequent formation of 4-hydroxy-2-nonenal (4-HNE), and prevented activation of apoptotic caspases and PARP-1 cleavage. Gene expression analyses showed that pioglitazone upregulated cochlear genes involved in both mitochondrial ROS production and detoxification pathways. The results of this study revealed that PPARs play important roles in the cochlea and demonstrate the potential of PPAR-targeting drugs as therapeutic agents for the treatment of hearing loss. Pioglitazone, a drug with a well-characterized safety and efficacy profile derived from > 27 million patient-years of use in the treatment of diabetes, appears to be an attractive candidate.

## Materials and methods

### Animal procedures

All animal procedures were conducted in compliance with the European Communities Council Directive of 24 November 1986 (86/609/EEC), and they were approved by the Kantonales Veterinäramt, Basel, Switzerland. Cochleae for culture studies were obtained from C57BL/6N mice on postnatal day 4 or 5. Cochleae used for immunohistochemistry were obtained from adult C57BL/6N mice (Harlan Ltd., Füllinsdorf Switzerland). Postnatal day 4 or 5 animals were sacrificed by decapitation while adult animals were sacrificed by an overdose of sodium pentobarbitol. Animals were maintained on a 12 h light/12 h dark schedule with free access to water and a standard mouse diet.

### Organ of Corti tissue culture and drug treatment

Organ of Corti (OC) explants were isolated according to previously described methods [11]. Briefly, animals were decapitated and cochlear microdissections were performed under a light microscope to isolate the OC and spiral ganglion. After isolation, OCs were incubated in culture medium (Dulbecco’s Modified Eagle Medium, supplemented with 30 U/mL penicillin, 1% N1 supplement, 10% fetal calf serum, 25 mM HEPES) at 37°C and 5% CO_2_, for 24 h. Next, PPAR agonists, dissolved in DMSO, were added (pioglitazone: 1 μM, 2 μM, 5 μM, 8 μM, and 10 μM; muraglitazar and tesaglitazar: 2 μM and 10 μM; fenofibric acid: 25 μM and 150 μM). Control OCs were incubated in culture medium with only DMSO (vehicle) and without drugs. Following a 24 h incubation with/without drugs, 50 μM gentamicin was added (Sigma-Aldrich, St. Louis, MO, USA) for an additional 24 h; the drugs remained present during exposure to gentamicin. The concentration of gentamicin (50 μM) was selected from pilot titration experiments conducted to define the concentration that reproducibly caused approximately 50% loss of HCs (S1 Fig).

### Hair cell counting and analysis

OCs were fixed in 4% paraformaldehyde (in PBS), permeabilized by washing in PBS-T (0.1% Triton X-100 in PBS), then stained in a 1:100 diluted solution of Alexa Fluor 488-labeled phalloidin (Molecular Probes, Eugene, USA) in PBS-T for 40 min at 4°C. After washing with PBS, OCs were mounted on a slide with Mowiol (Sigma-Aldrich, St. Louis, MO, USA) for microscopy. To evaluate the presence or absence of HCs, we scored the presence or absence of phalloidin-stained stereociliary bundles and circumferential F-actin rings on the cuticular plate of outer and inner HCs. Images were captured with a fluorescence microscope (Olympus IX71) equipped with an AxioCam system (Zeiss, San Diego, USA). The four rows of HCs were oriented longitudinally, and each sequential 0.20-mm field was scored for the presence of inner and outer HCs. For each individual OC, both outer HCs and inner HCs were counted in three randomly selected segments of the basal turn containing (60 outer HCs associated with 20 inner HCs) in a given microscopic field. These values were then averaged across the 20 OCs (N = 10 mice) used in each treatment condition. Data are expressed as the average number of HCs ± SD.

### Western blotting

After sacrifice by decapitation, cochleae were carefully microdissected in ice-cold PBS. Mouse brain and liver lysates were used as positive controls. OCs were placed in cell lysis buffer with a protease inhibitor cocktail (Sigma C3228, P8340), then homogenized for 1 min on ice. Protein concentrations were measured with NanoDrop 1000 (ThermoScientific). Samples were mixed with Laemmli sample buffer (Sigma S3401) and heated at 95°C for 5 min. Samples (10 μg protein per lane) were resolved on SDS-PAGE gels. After electrophoresis, proteins were blotted onto a polyvinylidene fluoride membrane. Non-specific sites were blocked with 5% non-fat dry milk dissolved in PBS-T for 1 h at room temperature. Membranes was washed with PBS-T (3 × 10 min), then incubated with primary antibodies in 5% non-fat dry milk dissolved in PBS. The following primary antibodies were used: rabbit polyclonal anti-PPARα (1:1000, ab8934), mouse monoclonal anti-PPARγ (1:1000, sc7273), mouse monoclonal anti-4-HNE (1:2000, ab48506), rabbit polyclonal anti-cleaved-caspase-3 (1:1000, sc22171), mouse monoclonal anti-PARP-1 (1:1000, sc74469) and mouse monoclonal anti-β-actin (1:2000, sc81178). After incubations with primary antibodies overnight at 4°C, membranes were washed with PBS-T (3 × 10 min) and incubated with an appropriate HRP-conjugated secondary antibody for 1 h at room temperature. After washing, bands were visualized with Super Signal West Dura Extended Duration Substrate (ThermoScientific). β-actin was used as control to demonstrate equivalent protein loading.

### Preparation of whole cochlear sections

Adult mice (3 months old) of both sexes were sacrificed with an overdose of sodium pentobarbital (100 mg/kg) and transcardially perfused with 50 ml of phosphate-buffered 4% paraformaldehyde (pH 7.4, 4° C). The inner ear was carefully removed, and decalcification was carried out in a light-protected flask for 10 days in a solution of 120 mM EDTA (Merck, New Jersey, USA) in distilled water (pH 6.8). The cochleae were then dehydrated in graded ethanol solutions (70%, 80%, 95%, and 3 × 100%, each for 1 h), xylol (3 × 1 h), and paraplast (2 × 1 h at -60°C and 1 × 10 h at -60°C). The samples were then embedded in paraffin at 56°C.

### Immunocytochemistry

For histological evaluation, 10-μm cochlear sections were cut on a Leitz microtome and mounted on Superfrost plus slides (Menzel, Braunschweig, Germany). Sections were deparaffinized, rehydrated, washed in PBS for 5 min, and processed for immunohistochemistry. Sections were incubated for 1 h at room temperature in blocking solution containing TBS (50 mM Tris, 0.9% NaCl), 0.5% Triton X-100 (pH 7.35), and 3% normal goat serum (NGS). Then, sections were incubated with primary anti-PPARγ or anti-PPARα antibodies (1:300) diluted in TBS with 1% NGS overnight at 4°C. After three washes in TBS, sections were incubated with the appropriate Alexa-conjugated secondary antibodies (1:250; Molecular Probes) diluted in TBS with 1% NGS for 2 h at room temperature. After washing in TBS, the sections were counterstained with DAPI and mounted on glass slides with Mowiol. Sections were visualized with an Olympus AX-70 microscope equipped with a digital camera. The recorded images were adjusted for brightness and contrast with Image-Pro Plus and Photoshop image processing software.

### RNA isolation and quantitative PCR

OCs intended for RNA isolation were stored in RNAlater (Ambion, USA). RNA was isolated with the Direct-Zol RNA MiniPrep kit (Zymo Research, USA). The quantity and quality of RNA was determined with a NanoDrop 1000 (ThermoScientific). The 260/280-nm absorbance ratios were between 1.8 and 2.1 for all samples. Total RNA (1000 ng) was reverse transcribed into cDNA with the High-Capacity cDNA Reverse Transcription Kit (Applied Biosystems, USA). Quantitative PCR was performed with an ABI Prism 7900HT Sequence Detection System (Applied Biosystems, USA) and with the Power Sybr Green Master Mix (Applied Biosystems, USA). The primer sequences used for quantitative PCR (qPCR) were: (5`to 3`): *Sod1 Forward-AAC CAG TTG TGT TGT CAG GAC*, *Reverse-CCA CCA TGT TTC TTA GAG TGA GG; Gpx1 Forward-CCA CCG TGT ATG CCT TCT CC*, *Reverse-AGA GAG ACG CGA CAT TCT CAA T; Cat Forward-GGA GGC GGG AAC CCA ATA G*, *Reverse-GTG TGC CAT CTC GTC AGT GAA; Ucp2 Forward-ATG GTT GGT TTC AAG GCC ACA*, *Reverse-TTG GCG GTA TCC AGA GGG AA; Hmox1 Forward-AAG CCG AGA ATG CTG AGT TCA*, *Reverse-GCC GTG TAG ATA TGG TAC AAG GA and Gapdh Forward-TGA CCT CAA CTA CAT GGT CTA CA*, *Reverse-CTT CCC ATT CTC GGC CTT G* (Microsynth, St. Gallen, Switzerland). The final primer concentration was 250 nM per reaction. The thermocycling parameters were: 10 min at 95° C, then 40 cycles of 95° C for 15 s and 60° C for 60 s. Template-free controls ensured that nonspecific amplification and DNA contamination could be excluded. The relative quantities of specifically amplified cDNAs were calculated with the comparative threshold cycle method, and *Gapdh* expression levels were used as the endogenous reference.

### Reactive oxygen species (ROS) detection

Cellular oxidative stress was measured in OC explants exposed for 24 h to 50 μM gentamicin with or without 10 μM Pioglitazone. OCs were incubated in 10 μM CellROX® Deep Red Reagent (Invitrogen, USA) diluted in medium at 37°C for 30 min, then co-stained with FITC-phalloidin, as described above. Images were captured with a fluorescence microscope (Olympus IX71, Japan) equipped with an AxioCam system (Zeiss, San Diego, USA) and analyzed with Fiji-win 32 software. A fixed region of interest (ROI) was defined for all images (20 images per treatment condition), and signal intensity vs. background was quantified. Brightness was calibrated in the range of 0–255 arbitrary units [12, 13].

### Caspase assay

Apoptosis was determined with a caspase detection kit, Caspatag Pan-Caspase, (Millipore/Chemicon, Germany). In this system, green fluorescence intensity is proportional to the amount of active caspase. Staining was performed according to the manufacturer’s instructions, followed by co-staining with Rhodamine-phalloidin, as described above. Images were processed and analyzed with Fiji-win 32 software. The green signal intensity was measured and the background was subtracted. The defined ROI was the same for all images. The brightness was calibrated in the range of 0–255 arbitrary units.

### Glutathione assay

The levels of reduced and oxidized glutathione were determined with the GSH/GSSG Ratio Detection Assay Kit (Abcam, UK), according to the manufacturer`s instructions. Absorbance measurements were performed with a Synergy H1 reader (BioTek). Values are presented as the ratio of reduced:oxidized glutathione (GSH/GSSG).

### Statistical analysis

The statistical software, GraphPad Prism 7 (La Jolla, CA, USA), was used to analyze data. Unless otherwise noted, data are presented as the mean ± SD. Treatment effects were analyzed with the parametric Sudent`s t test. In cases where more than two groups were compared, one-way analysis of variance (ANOVA) followed by Bonferroni *post*-test was performed. P-values <0.05 were considered statistically significant.

## Results

### PPARγ and PPARα are expressed in the inner ears of neonatal and adult mice

**Fig 1:**
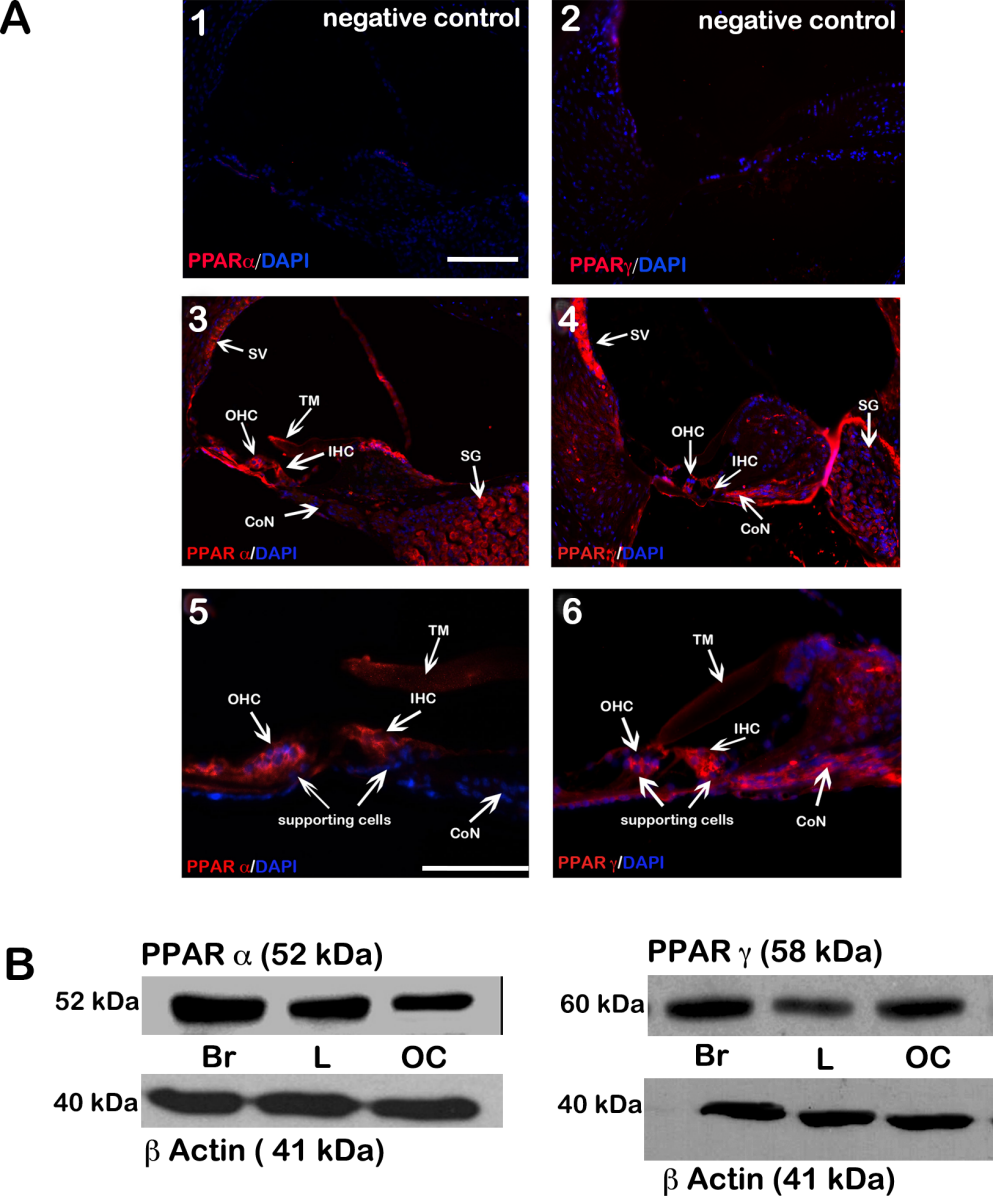
Expression and localization of PPARγ and PPARα proteins in mouse cochlea. (A) Fluorescence micrographs of adult mouse cochlear sections stained for PPARα and PPARγ. Negative controls (*panels 1 and 2*), red immunostaining for PPARα (*panels 3 and 5*) and for PPARγ (*panels 4 and 6*). Scale bar: 50 μm. DAPI: nuclear stain (blue). (B) Western blots of protein extracts show (*left*) PPARα and (*right*) PPARγ levels in the organ of Corti (OC) from 5-day old neonatal mice. ß-actin was used as a loading control. OC, organ of Corti; Br, mouse brain; L, mouse liver. kDa, kilodalton, TM, tectorial membrane; DAPI, 4',6-diamidino-2-phenylindol.

We first investigated the expression and distribution of PPARγ and PPARα in the mouse cochlea. Specific antibodies against PPARγ and PPARα were used to stain formalin-fixed paraffin-embedded adult mouse cochlear sections. Both PPARγ and PPARα were found in inner and outer HCs, in the stria vascularis, spiral ganglion and cochlear nerve. Higher magnification images reveal the presence of PPARγ in supporting cells and cells of the basal lamina; in contrast, little PPARα staining was detected in these cell types (Fig 1A). Western blot analysis of proteins isolated from OCs from 5-day old mice confirmed that PPARγ and PPARα were also expressed in the sensory epithelium of the neonatal mouse cochlea (Fig 1B). The levels of PPARγ and PPARα proteins in the organ of Corti were qualitatively similar to levels in mouse brain and liver, where PPARγ and PPARα have been well characterized.

### Pioglitazone and other diverse PPAR agonists protect from gentamicin—induced hair cell death

**Fig 2:**
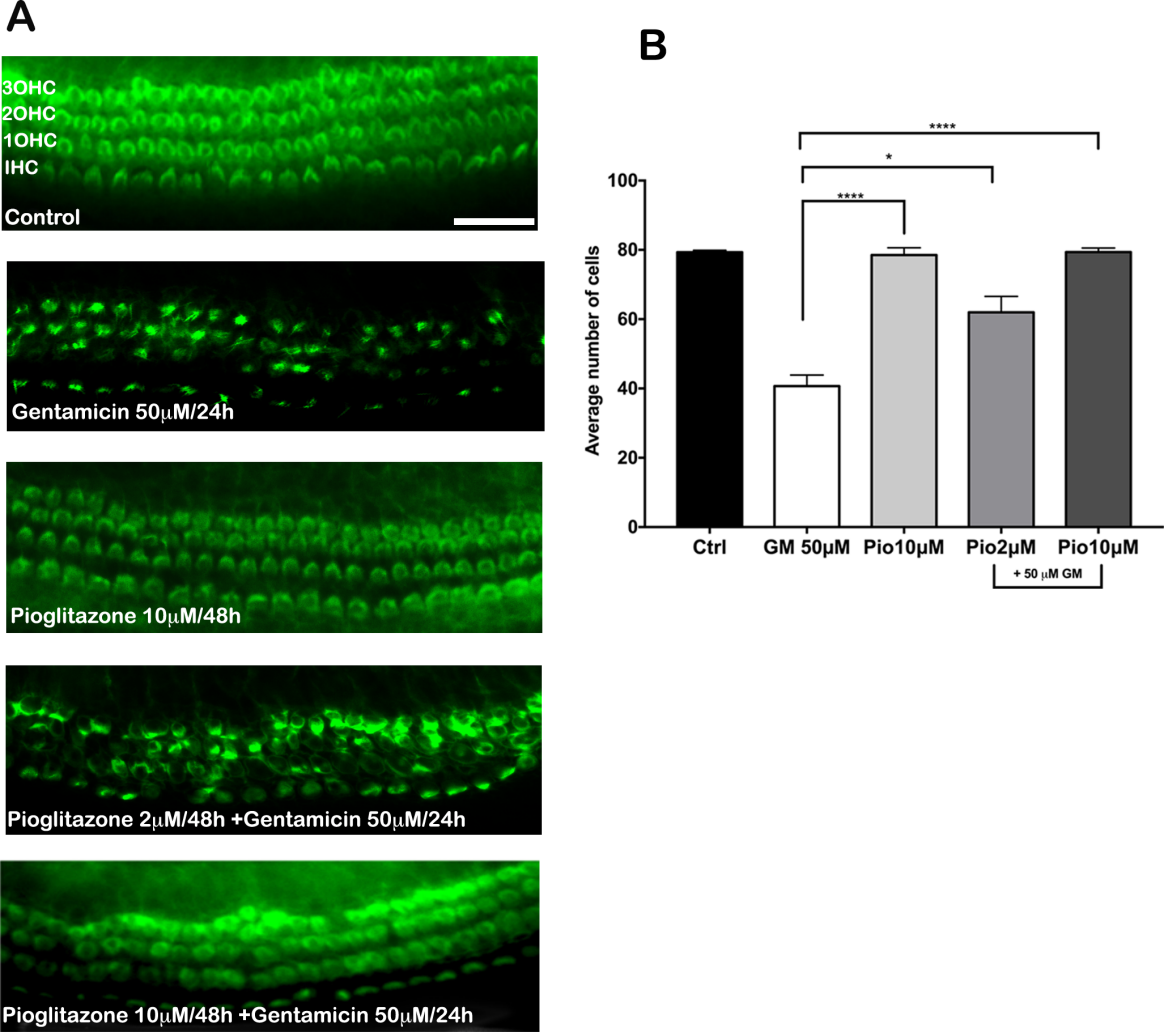
Pioglitazone prevented gentamicin (GM)-induced hair cell death in mouse organ of Corti (OC) explants. (A) Representative fluorescence micrographs of the basal turn of OCs show auditory hair cells detected with Alexa Fluor 488-phalloidin (green). OC were incubated in the following conditions (*top to bottom*): medium alone for 48 h; medium 24 h, then GM (50 μM) for 24 h; pioglitazone (10 μM) alone for 48 h; and pioglitazone at 2 or 10 μM for 48 h, with GM (50 μM) added for the last 24 h. Scale bar: 50 μm. (B) Quantitative analysis of hair survival. N = 20 explants per group; ns (not significant), *p<0.05 and ****p<0.0001 compared to GM treatment alone. Data are the mean number of surviving hair cells ± SD. OHC, outer hair cell; IHC, inner hair cell.

**Fig 3:**
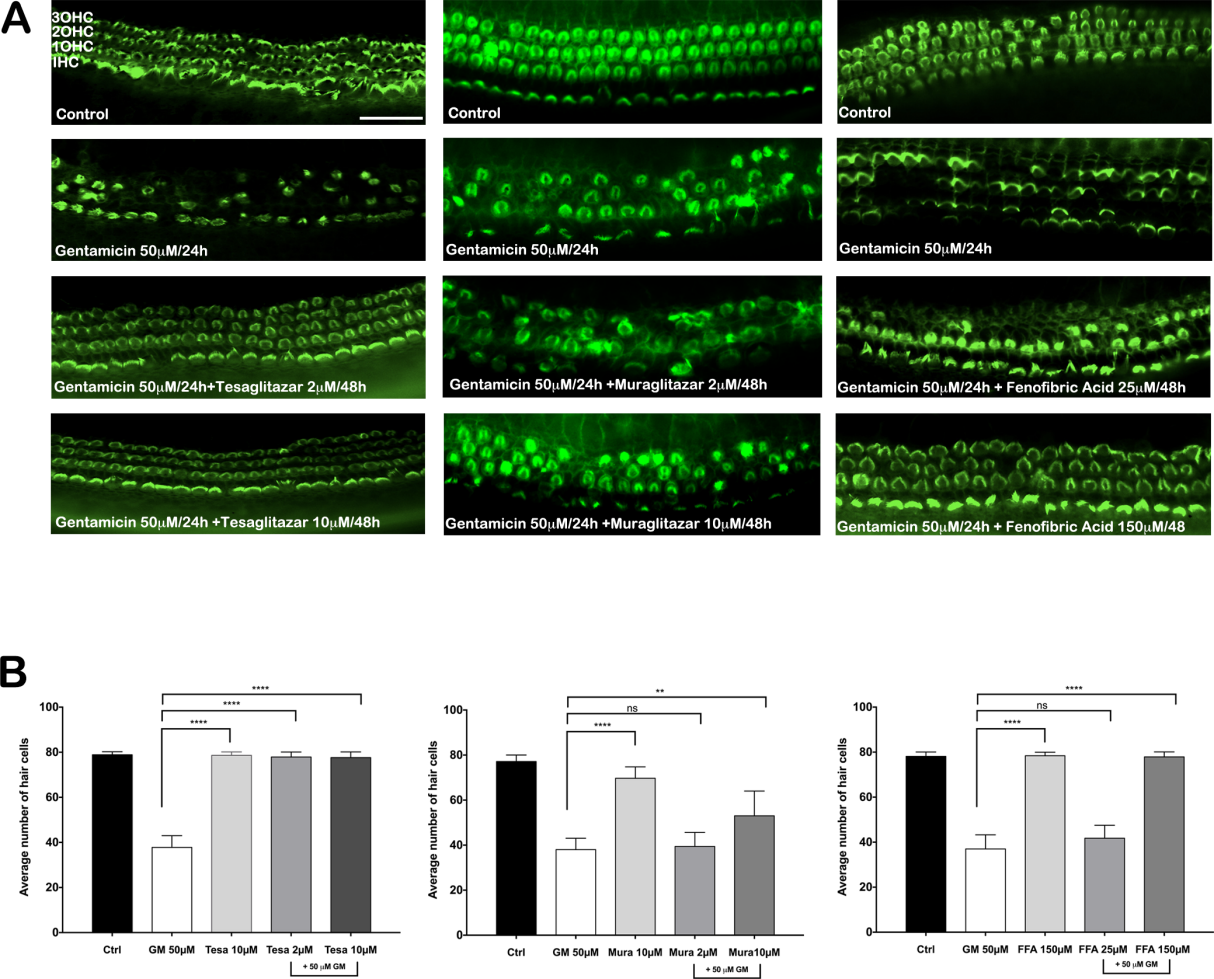
Structurally diverse PPAR agonists prevented gentamicin (GM)-induced hair cell death in mouse organ of Corti (OC) explants. (A) Representative fluorescence micrographs of the basal turn of OCs show auditory hair cells stained with Alexa Fluor 488-phalloidin (green) and counted under a fluorescence microscopy. OCs were incubated in the following conditions (*from top to bottom in each column*): medium alone (Control) for 48 h, medium 24 h, then PPAR agonist (two concentrations) for 48 h, with GM (50 μM) added for the last 24 h; the PPAR agonists were: (*left*) tesaglitazar (2 or 10 μM), (*middle*) muraglitazar (2 or 10 μM), and (*right*) fenofibric acid (25 or 150 μM). Scale bar: 50 μm. (B) Quantitative analysis of hair cell survival. N = 20 explants per group. **p<0.01 and ****p<0.0001, compared to GM treatment alone. Data are the mean number of surviving hair cells ± SD. OHC = outer hair cell; IHC = inner hair cell.

To assess the potential roles of PPARs in gentamicin-induced HC death, we evaluated the effects of pioglitazone and several diverse PPARγ and PPARα agonists (pioglitazone: PPARγ -selective; fenofibric acid: PPARα-selective; and tesaglitazar and muraglitazar: PPARγ/α dual agonists) to prevent gentamicin toxicity in cultured OC explants. OCs from 5-day old neonatal mice were incubated in culture for 48 h in the presence of the selected PPAR agonists. First, we found that neither pioglitazone nor the other agents alone had any effect on HC number or morphology (Figs 2 and 3), which indicated that these drugs were not toxic. In cultures treated only with gentamicin, approximately 50% of HCs were lost, as reflected by the absence of phalloidin-stained stereociliary bundles and circumferential F-actin rings (Figs 2A and 3A). Pioglitazone and the other PPAR agonists strongly inhibited gentamicin-induced HC death (Figs 2 and 3); the highest concentrations of pioglitazone, tesaglitazar, and fenofibric acid completely prevented gentamicin-induced HC loss (Figs 2B and 3B; ****p<0.0001). The dual PPARγ/α agonist, muraglitazar, was only partially protective. The effective concentrations that prevented gentamicin-induced HC loss were roughly consistent with the reported EC_50_ values of each compound for activating PPARγ and PPARα transcriptional activities in cell-based assays [14]. We determined a complete dose-response for pioglitazone (S2 Fig). Concentrations of pioglitazone above 0.5 μM significantly prevented gentamicin toxicity, and maximum protection was achieved at concentrations >8 μM (****p<0.0001). We also showed that a regimen in which OCs were treated with pioglitazone added at the same time as gentamicin, significantly prevented gentamicin-induced hair cell loss (S3 Fig). No toxicity of pioglitazone alone was observed at concentrations as high as 50 μM (S4 Fig).

**Fig 4:**
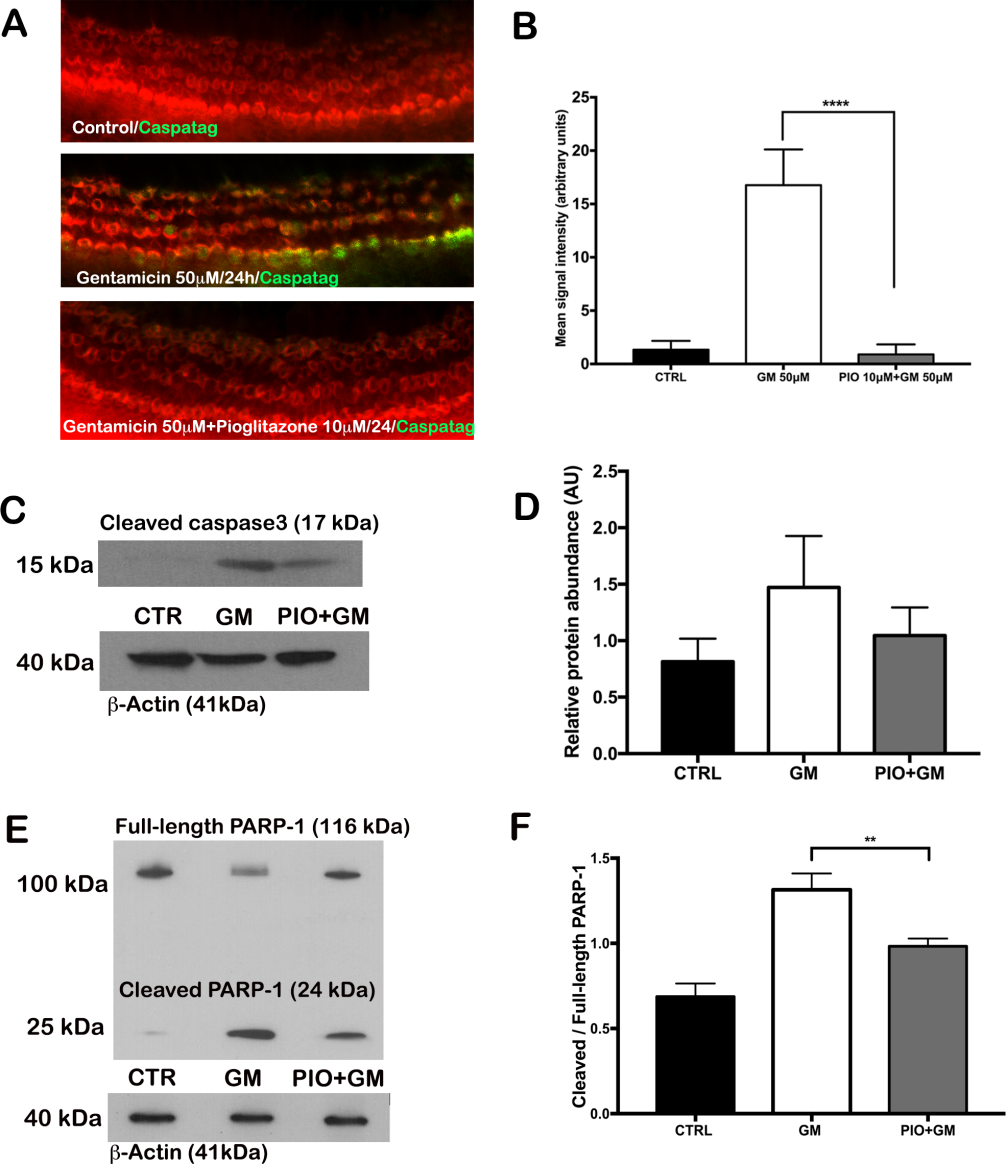
Pioglitazone (PIO) prevented gentamicin (GM)-induced activation of pro-apoptotic caspases in mouse organ of Corti. (A) Representative fluorescence micrographs of the basal turn of OCs show auditory hair cells detected with rhodamine-phalloidin (red), and activated caspases detected with Caspatag (green). Mouse OCs were treated as described in Fig 2, without (control, CTRL) or with GM +/- PIO. Scale bar: 50 μm (B) Quantification of the fluorescent signal in panel A. N = 10 explants per group; ****p <0.0001. (C) Representative Western blot shows the cleavage product of caspase 3 in lysates from OCs treated without (CTR) or with GM (50 μM) or GM + PIO (10 μM); ß-actin is the loading control. (D) Quantification of Western blots for the caspase 3 cleavage product in Panel C. The values shown indicate the mean expression levels normalized to ß-actin. N = 3; (E) Representative Western blot shows full-length PARP-1 and its cleaved 24kDa fragment in lysates from hair cells treated without (CTR) or with GM (50 μM) or GM with PIO (10 μM). (F) Quantification of Western blot signals represented by ratio of signals cleaved/full-length PARP-1 normalized to ß-actin. N = 3; **p<0.01.

To confirm that HC loss occurred by apoptosis, we performed assays to detect activated effector caspases in OC explants cultured with gentamicin in the presence or absence of 10 μM pioglitazone. Viable HCs were identified with rhodamine phalloidin staining, and active caspases were evaluated with a Caspatag assay by fluorescence microscopy. No signal for activated caspases was detected in control OCs (Fig 4). A large number of HCs were positive for activated caspases after gentamicin treatment, which suggested that HCs died through apoptosis (Fig 4A and 4B). Pioglitazone nearly completely prevented caspase activation by gentamicin in this assay, as reflected by signal levels similar to those in control, untreated OC’s. We also performed Western blots of OC lysates to detect cleaved caspase 3. Consistent with the fluorescent results, gentamicin increased the formation of cleaved caspase 3, which was partially opposed by pioglitazone (non-significant) (Fig 4C and 4D). Finally, we evaluated the levels of the caspase substrate PARP-1. Gentamicin induced significant increase in PARP-1 cleavage which was significantly inhibited by treatment with pioglitazone (Fig 4E and 4F). These results together demonstrated that pioglitazone protected HCs by blocking gentamicin-induced apoptosis.

### Pioglitazone inhibits gentamicin-induced ROS production and 4-HNE formation

**Fig 5:**
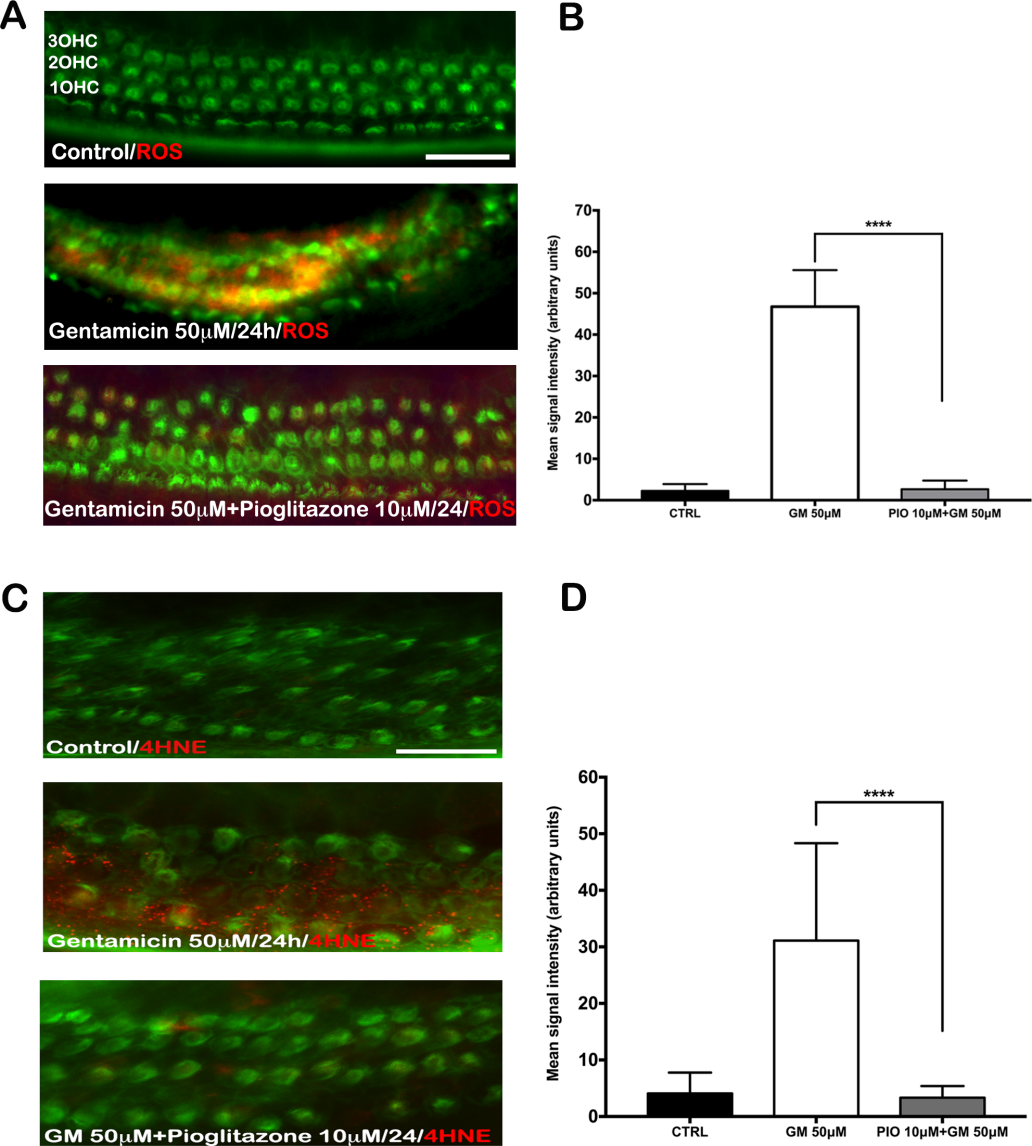
Pioglitazone (PIO) inhibited the production of reactive oxygen species (ROS) and the lipid peroxidation product, 4-hydroxy-2-nonenal (4-HNE). (A-D) Mouse OCs were treated as described in Fig 2 with gentamicin (GM) +/- PIO. Representative micrographs from the basal turn of the organ of Corti were stained with Alexa Fluor 488-phalloidin (green) and either (A) the ROS indicator, CellRox (red) or (C) the 4-HNE antibody (red). Scale bar: 50 μm. (B, D) Quantification of signal intensities. GM strongly induced ROS production and lipid peroxidation. Both effects were nearly completely prevented by PIO. Values are the mean ± SD (N = 10 explants per group; ****p <0.0001).

PPARs are involved in several pathways that maintain cellular oxidative balance, including the ROS production/detoxification pathway, NF-kappa B signaling, c-Jun N-terminal kinase signaling, and the Akt/PI3K pathway. Cellular redox status is commonly assessed by tracking the formation of superoxide and lipid peroxidation markers, such as 4-HNE or isoprostane. We observed that gentamicin induced a marked increase in both cellular ROS (Fig 5A and 5B) and 4-HNE in mouse organ of Corti explants, evident with fluorescence microscopy (Fig 5C and 5D) and with Western blot analysis (S5 Fig). These effects of gentamicin were nearly completely blocked by pioglitazone. These results indicated that one of pioglitazone’s key mechanisms in protecting HCs from gentamicin-induced apoptosis was to reduce ROS levels in the sensory epithelium of the cochlea.

### Pioglitazone restores HC antioxidant capacity by regulating expression of endogenous redox enzymes

**Fig 6:**
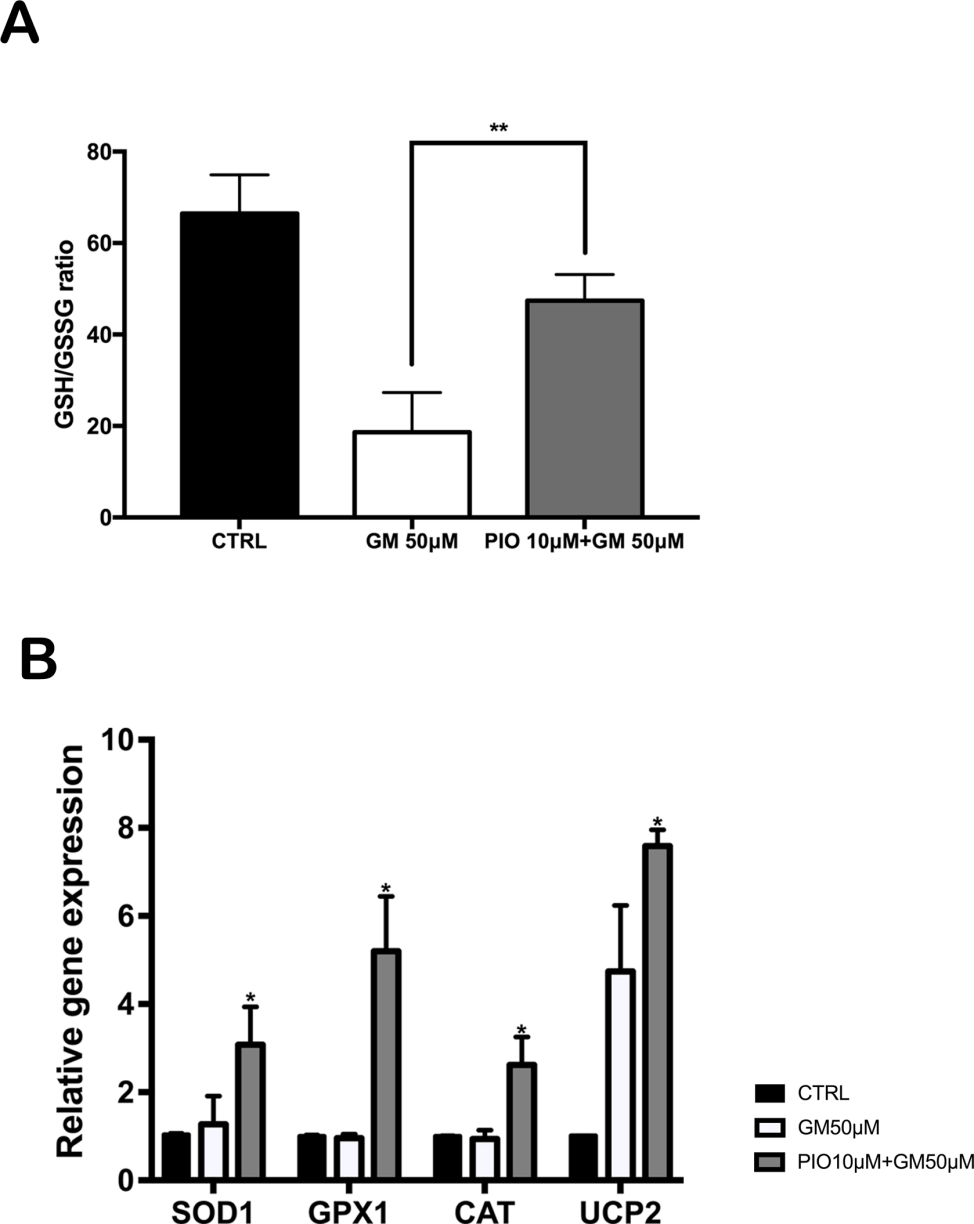
Pioglitazone (PIO) restored the redox balance in mouse OCs after exposure to gentamicin (GM). Mouse OCs were treated as described in Fig 2 with GM +/- 10 μM PIO. (A) GM caused depletion of endogenous antioxidants, as reflected by a 75% reduction in the ratio of reduced:oxidized glutathione (GSH/GSSG). (B) GM alone had no effect on the relative expression of *Sod1*, *Gpx1*, or *Cat*, but upregulated *Ucp2* expression. Pioglitazone upregulated the expression of all four genes, including *Ucp2* expression, which correlates with significant improvement in the cellular redox state as reflected in panel A. Results are the mean fold-change in transcript levels ± SD. N = 3; *p<0.05; **p<0.01, compared to untreated OCs.

Oxidative stress occurs as a consequence of excess ROS production, antioxidant depletion, or combination of both factors. To investigate the molecular mechanisms by which pioglitazone prevents gentamicin-induced ROS formation, we determined antioxidant capacity by measuring GSH/GSSG ratio in OC explants after gentamicin and pioglitazone exposure [15]. Gentamicin-treated OCs were strongly depleted in GSH, as indicated by the profound reduction in the GSH/GSSG ratio, to approximately 25% of the ratio observed in untreated OCs (Fig 6A). Pioglitazone opposed this effect, resulting in restoration of the GSH/GSSG ratio to about 70% of normal (Fig 6A).

We next measured mRNA expression levels of superoxide dismutase (*Sod1*), glutathione peroxidase (*Gpx1*), and catalase (*Cat*). Gentamicin had no effect on these mRNA levels, but pioglitazone significantly upregulated all three mRNAs in the presence of gentamicin, by approximately 3-5 fold (Fig 6B). We also measured uncoupling protein 2 (*Ucp2*). Pioglitazone has been shown to protect the heart by upregulating expression of UCP2 to reduce ROS production during ischemia-reperfusion injury [16, 17]. We found that *Ucp2* mRNA is expressed in the organ of Corti (Fig 6B). Gentamicin upregulated *Ucp2* expression by approximately 5-fold, which was further increased to approximately 8-fold by addition of pioglitazone. These results together, suggest that upregulation of endogenous antioxidant pathways by pioglitazone prevents gentamicin-induced oxidative stress and HC apoptosis by both reducing ROS production and potentiating ROS detoxification.

**Fig 7:**
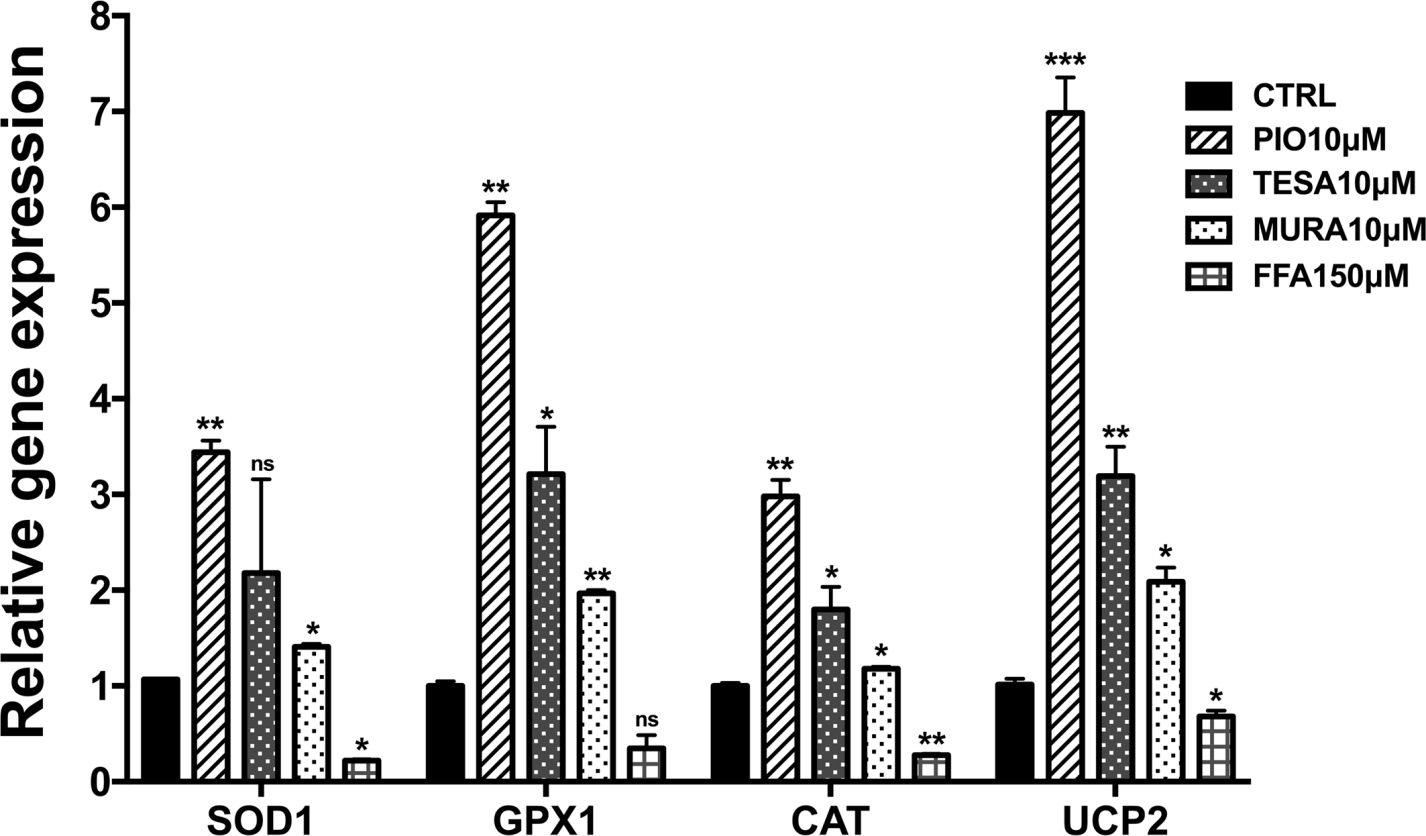
Effects of structurally diverse PPAR agonists on redox gene expression in mouse organ of Corti (OC) explants. Pioglitazone (PIO), muraglitazar (MURA) and tesaglitazar (TESA) significantly induced expression of of superoxide dismutase (*Sod1*), glutathione peroxidase (*Gpx1*), catalase (*Cat*), and uncoupling protein 2 (*Ucp2*), while fenofibric acid (FFA) repressed their expression. Results are the mean fold-change in transcript levels ± SD. N = 3; ns (not significant); *p<0.05; **p<0.01; ***p<0.001, compared to untreated OCs.

We then compared the effects of pioglitazone to other PPAR agonists (tesaglitazar, muraglitazar, and fenofibric acid) on regulation of the panel of redox-related genes (*Sod1*, *Gpx1*, *Cat*, and *Ucp2*). Pioglitazone alone showed a similar pattern of upregulation as was observed in experiments in the presence of gentamicin. Tesaglitzar and muraglitazar we able to upregulate expression of all four genes but to a lesser extent than pioglitazone. In contrast to the effects of the PPARγ (PIO) and PPARγ/α dual agonists (TESA and MURA), the PPARα-selective agonist, fenofibric acid (FFA), modestly downregulated expression of all four genes (Fig 7). These divergent effects on expression of *Sod1*, *Gpx1*, *Cat* and *Ucp2* in the organ of Corti suggest that PPARγ and PPARα activate distinct mechanisms. In order to investigate this further, we compared the effects of FFA (PPARα-selective) vs. PIO (PPARγ-selective) on *Hmox1* expression in OC’s. We found that both agonists significantly induced *Hmox1* expression, by approximately 15-fold for PIO and 150-Fold for FFA (S6 Fig). These data together, demonstrate protective roles for both PPARγ and PPARα in the organ of Corti, but reveal that these related transcription factors affect divergent gene networks and antioxidant pathways.

## Discussion

A recurring theme in hearing loss is the central role played by cellular redox imbalances in the cochlea. The mechanisms that lead to hearing loss caused by diverse insults (e.g., aminoglycoside antibiotics, anticancer drugs, noise exposure, and aging) are not completely understood. However, it has been demonstrated that all causes lead to oxidative stress, which ultimately influences the balance between auditory HC survival and apoptosis [18–21].

Well known as drug targets, the PPARs comprise a family of three ligand-regulated transcription factors involved in many physiological and pathological processes (lipid metabolism, type 2 diabetes, atherosclerosis, and inflammation) [22, 23]. Anti-diabetic drugs in the thiazolidinedione class, including pioglitazone, bind to and activate the transcriptional activity of PPARγ, which results in improvements in insulin signaling and metabolic function. Drugs in the fibrate class, including fenofibrate, bind to PPARα and primarily affect plasma lipid levels. Downstream of their metabolic effects, these agents ameliorate cellular oxidative/nitrosative stress through several pathways that regulate cellular oxidative balance, including the ROS production pathway, NF-kappaB signaling, c-Jun N-terminal kinase stress-responsive signaling, and the Akt/Pi3K pathway [24, 25]. PPAR agonists also display anti-inflammatory and neuroprotective effects, mediated by reducing proinflammatory cytokines and modulating NMDA excitotoxicity [26]. PPARγ agonists have also shown significant favorable effects in models of neurological diseases [27–29]. Together, these findings suggest that PPAR agonists could be effective therapeutic agents in preventing hearing loss that arises from various etiologies.

No previous reports have described the roles and expression of PPARs in the inner ear. By Western blot analysis, we showed that PPARα and PPARγ proteins are present at levels in the cochlea qualitatively similar to levels in brain and liver. Immunostaining results indicated that both PPARα and PPARγ are expressed in diverse structures of the cochlea, including both inner and outer auditory HCs. PPARγ is expressed in the spiral ganglion neurons while PPARα is absent.

Gentamicin causes HC loss by affecting mitochondrial metabolism leading to increased production of reactive oxygen species [30]. We found that pioglitazone, as well as diverse dual and specific PPAR agonists provided partial to complete protection of auditory HCs from gentamicin toxicity. In further experiments, we found that gentamicin dramatically increased cellular superoxide levels, which led to increased formation of 4-HNE, a toxic lipid product, and activation of pro-apoptotic caspases and PARP-1 cleavage. Pioglitazone opposed these effects by nearly completely blocking the formation of ROS and 4-HNE. By opposing gentamicin-induced oxidative stress, pioglitazone effectively prevented caspase activation, PARP-1 cleavage, and HC apoptosis.

To understand the potential mechanisms by which pioglitazone prevented gentamicin-induced oxidative stress, we examined the effects of pioglitazone on the antioxidant system in the organ of Corti. The cellular antioxidant defense system consists of multiple enzymes involved in ROS formation and metabolism. GSH is a thiol-containing tripeptide that acts as a cellular scavenger of oxygen-free radicals. GSH-regulatory enzymes, including SOD-1, catalase, and GSH reductase, are expressed in the cochlea. We found that exposing cultured OCs to gentamicin resulted in a drastic depletion of the endogenous antioxidant pool, reflected by a strong reduction in the GSH/GSSG ratio, and that pioglitazone restored this ratio. In OCs, agonists that possess PPARγ activity (pioglitazone, tesaglitazar, and muraglitazar) significantly upregulated the expression of *Sod1*, *Gpx1*, *Cat*, and *Ucp2*. Interestingly, we found that the PPARα agonist fenofibric acid significantly repressed mRNA expression of these genes. These results contrast with those of a recent report, which found that fenofibric acid nevertheless prevented SOD-1 and catalase protein depletion and opposed ROS and hair cell apoptosis induced by gentamicin in the OC, consistent with our results [31]. Further data pointed to a role of heme oxygenase-1 (HO-1) as a major mediator of the antioxidant effects downstream of PPARα. Consistent with the reported data on HO-1 protein levels in cochlea, we show that both PPARγ and PPARα-selective agonists (pioglitazone and fenofibric acid, respectively) significantly induced the expression of *Hmox1* mRNA in OCs [31]. Based on the observation that pioglitazone, tesaglitazar, and muraglitazar all share PPARγ activity, and that fenofibric acid is PPARα-selective, these data suggested that PPARγ and PPARγ/α dual agonists may be more effective than PPARα agonists for potentiating antioxidant gene expression in the organ of Corti.

Furthermore, we found that all agonists possessing PPARγ activity (pioglitazone, muraglitazar, and tesaglitazar) but not the PPARα agonists fenofibric acid significantly upregulated the expression of *Ucp2* in the OC. As a target of PPARγ, UCP2 has gained appreciation for its role in opposing the production of mitochondria-derived ROS [17, 32]. In mouse heart, pioglitazone protects cardiomyocytes from ischemic-reperfusion injury by upregulating *Ucp2*, leading to mild depolarization of the inner mitochondrial membrane potential leading to a reduction in superoxide levels [16]. Several additional reports have confirmed that UCP2 plays a role in preventing oxidative-stress-induced tissue damage [33–35]. Finally, it has been shown that *Ucp2* overexpression could extend the lifespan of *Sod-2* knockout mice exposed to systemic oxidative stress [36]. Our findings suggest that UCP2 may be an additional mechanism by which PPARγ agonists specifically, such as pioglitazone, may protect auditory HCs from oxidative stress.

In summary, we have shown that the peroxisome proliferator-activated receptors α and γ are expressed in the cochlea and play distinct roles in the cochlear response to oxidative stress, which has become appreciated as a common mechanism for hearing loss of all causes. Our data, which demonstrate that drugs such as pioglitazone affect multiple pathways of cochlear protection, support the potential of these agents as treatments for most types of hearing loss. In particular, pioglitazone has a well established safety and efficacy profile, derived from more than 17 years of clinical use as an oral anti-diabetic agent. The dual agonists muraglitazar and tesaglitazar were terminated due to safety concerns in development and never reached the market. Moreover, pioglitazone is the only approved PPARγ agonist with significant ability to cross the blood-brain barrier [37, 38] and has demonstrated neuroprotective benefits in models of Alzheimer’s disease, Parkinson’s disease, epilepsy, and stroke [39–42].

A key implication of these results is the possibility that pioglitazone offers an alternative to targeting oxidative stress that offers advantages vs. the many antioxidant agents that have been explored for hearing loss (e.g. d-methionine, ebselen, n-acetylcysteine). These agents must be administered in high doses, have poor physicochemical properties, and chemically detoxify only a limited spectrum of reactive oxygen and reactive nitrogen species. Pioglitazone, on the other hand, is highly effective to potentiate the endogenous glutathione system in the cochlea, bolstering the innate ability of cochlear cells to inactivate free radicals. In addition, pioglitazone, by induction of UCP2, may also favorably intervene at the level of mitochondrial ROS production. The ability to stimulate innate antioxidant defenses of the cochlea with pioglitazone appears to offer an attractive alternative to antioxidant therapy for hearing loss.

## Supporting information

S1 Fig Gentamicin titration for establishing the concentration that induces approximately 50% HC loss. (TIF)

S2 Fig Dose dependent effect of pioglitazone on HC survival. Dose-response assay shows that the effect of gentamicin (GM) on hair cell death depends on the pioglitazone (PIO) concentration in mouse organ of Corti (OC) explants. Auditory hair cells were stained with Alexa Fluor 488-phalloidin and counted under fluorescence microscope. OC were incubated with either medium alone for 48 h (CTRL), medium for 24 h then GM (50 μM) for 24 h, or a range of PIO concentrations (from 0.5 to 10 μM) for 48 h, then GM (50 μM) added for the last 24 h. GM treatment caused >50% loss of hair cells. PIO at concentrations >1 μM protected hair cells from GM toxicity. **p<0.01 and ****p<0.0001, compared to GM treatment alone. Data are the mean number of surviving hair cells ± SD.
(TIF)

S3 Fig Hair cell survival without pioglitazone pretreatment. OC were incubated in the following conditions medium alone for 48 h; medium 24 h, then GM (50 μM) for 24 h; medium for 24h then pioglitazone (10 μM) with GM (50 μM) for the last 24 h. N = 5 explants per condition; ****p<0.0001. Data are the mean number of surviving hair cells ± SD. OHC, outer hair cell; IHC, inner hair cell.
(TIF)

S4 Fig Pioglitazone at a high concentration of 50 μM shows no toxicity in phalloidin stained OC culture. (TIF)

S5 Fig Western blot of OC protein extracts probed with the 4-HNE antibody. Western blot shows 4-hydroxy-2-nonenal (4-NHE)-modified proteins extracted from mouse organ of Corti (OC). Explanted OCs were untreated (CTR) or exposed to gentamicin (GM), either alone or with pioglitazone (PIO+GM). The blot was probed with the 4-HNE-specific antibody. The results indicate that gentamicin increased 4-HNE modifications, and the addition of pioglitazone prevented 4-HNE damage induced by gentamicin. ß-actin was used as a loading control.
(TIF)

S6 Fig Fenofibric acid (150 μM) and pioglitazone (10 μM) are increasing *Hmox1* expression in mouse OCs. (TIF)
