# Supplementary figures and images for: Effects of peroxisome proliferator activated receptors (PPAR)-γ and -α agonists on cochlear protection from oxidative stress
Source: PLoS One. 2017 Nov 28;12(11):e0188596. doi: 10.1371/journal.pone.0188596 (PMC5705132; doi:10.1371/journal.pone.0188596)

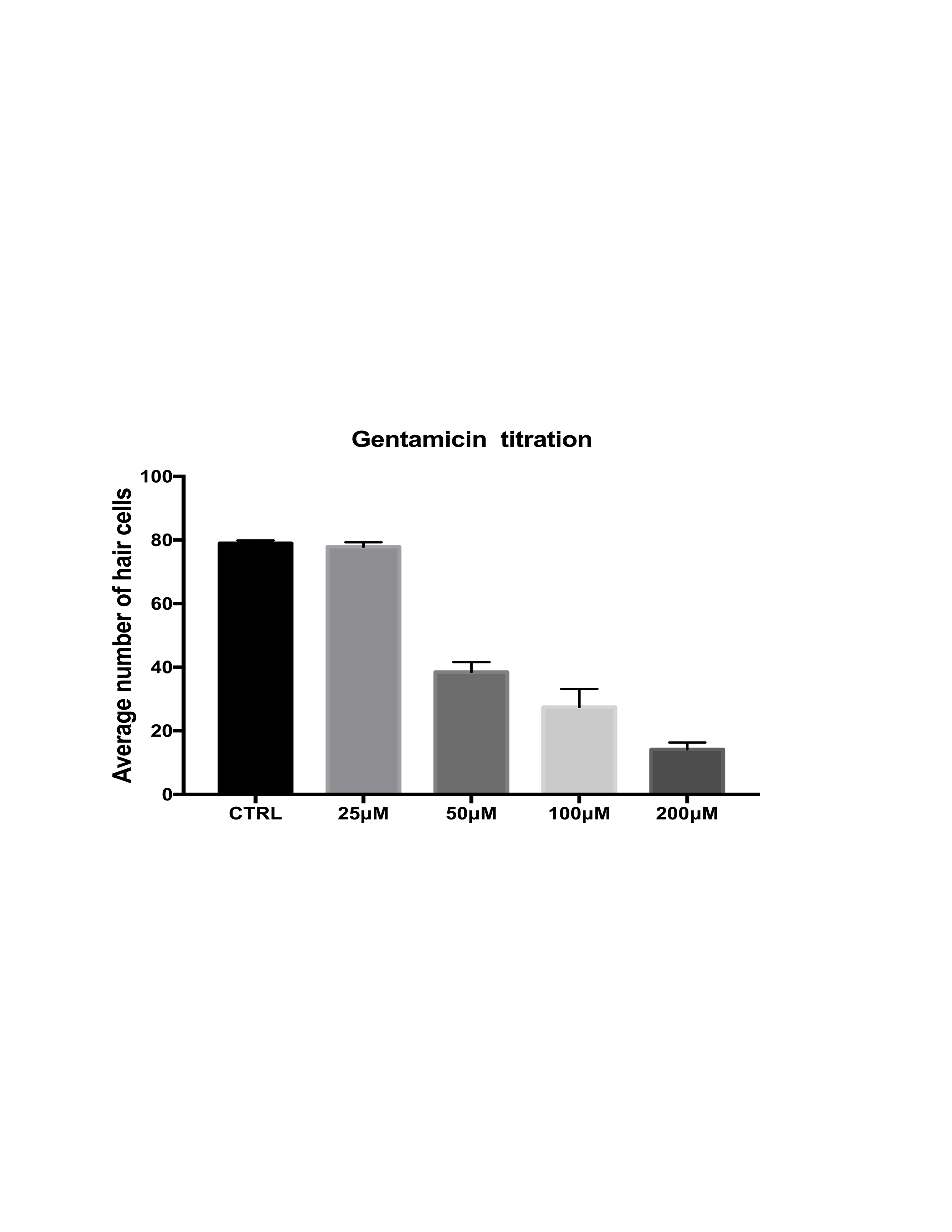

Supplement: S1 Fig — (TIF) [file pone.0188596.s001.tif]

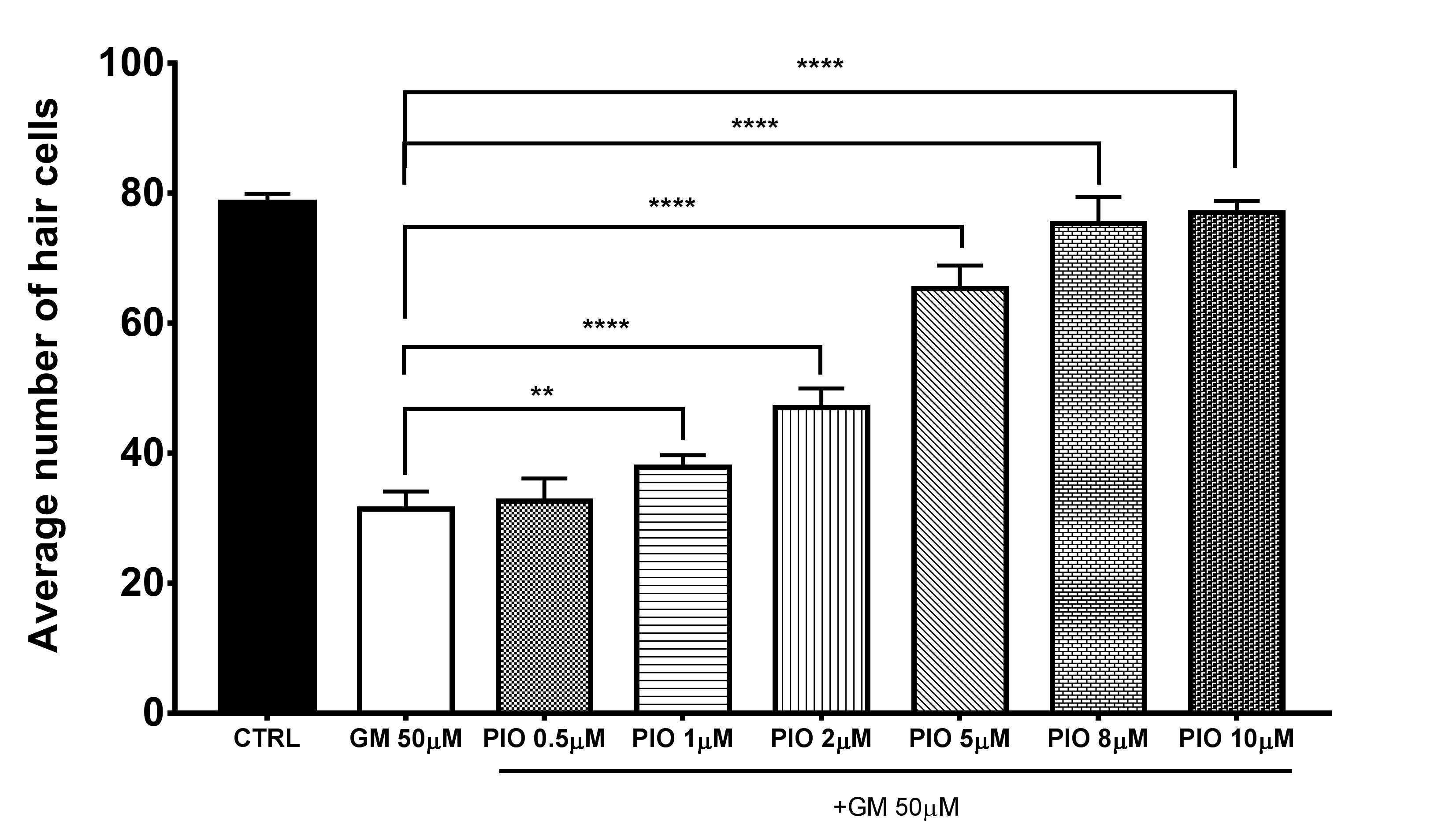

Supplement: S2 Fig — Dose-response assay shows that the effect of gentamicin (GM) on hair cell death depends on the pioglitazone (PIO) concentration in mouse organ of Corti (OC) explants. Auditory hair cells were stained with Alexa Fluor 488-phalloidin and counted under fluorescence microscope. OC were incubated with either medium alone for 48 h (CTRL), medium for 24 h then GM (50 μM) for 24 h, or a range of PIO concentrations (from 0.5 to 10 μM) for 48 h, then GM (50 μM) added for the last 24 h. GM treatment caused >50% loss of hair cells. PIO at concentrations >1 μM protected hair cells from GM toxicity. **p<0.01 and ****p<0.0001, compared to GM treatment alone. Data are the mean number of surviving hair cells ± SD. (TIF) [file pone.0188596.s002.tif]

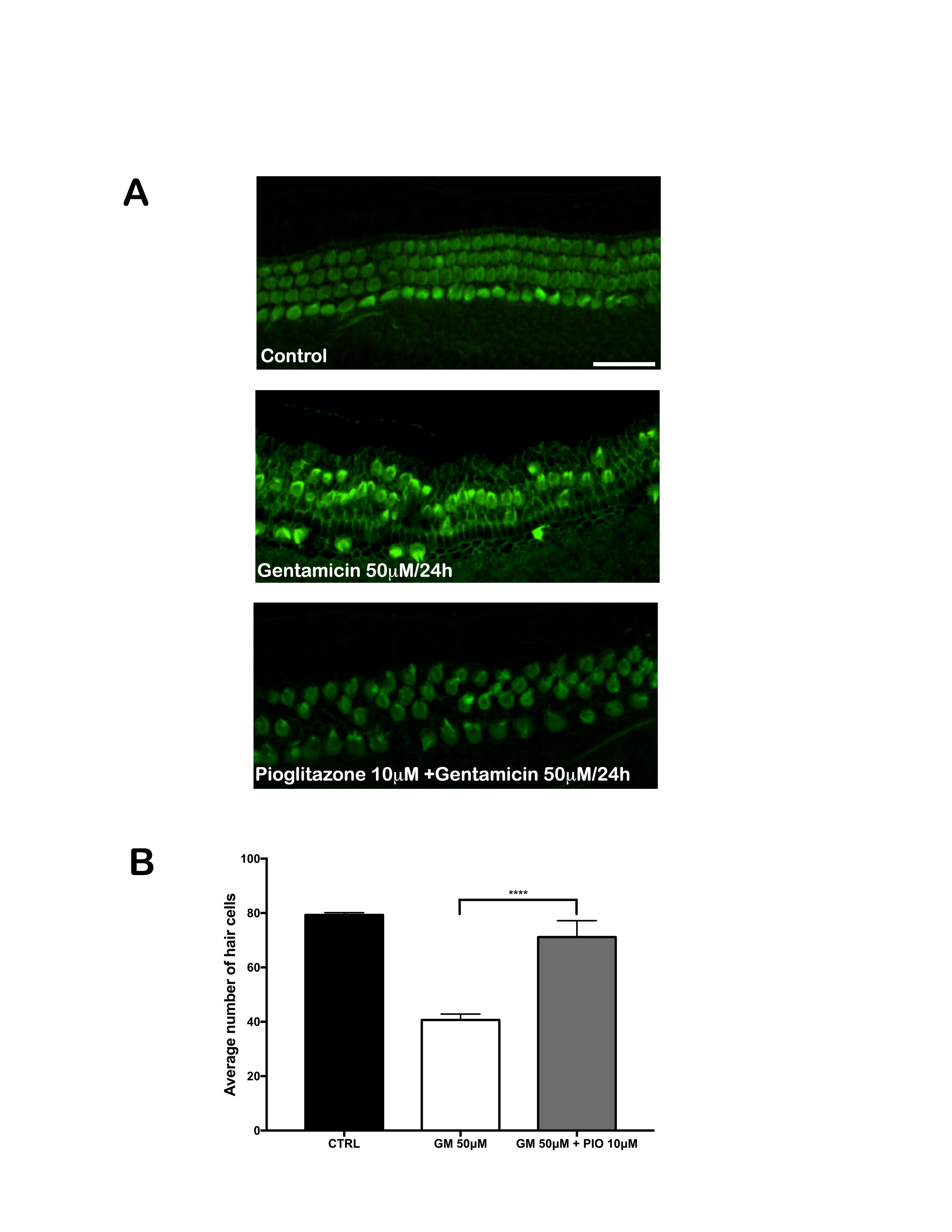

Supplement: S3 Fig — OC were incubated in the following conditions medium alone for 48 h; medium 24 h, then GM (50 μM) for 24 h; medium for 24h then pioglitazone (10 μM) with GM (50 μM) for the last 24 h. N = 5 explants per condition; ****p<0.0001. Data are the mean number of surviving hair cells ± SD. OHC, outer hair cell; IHC, inner hair cell. (TIF) [file pone.0188596.s003.tif]

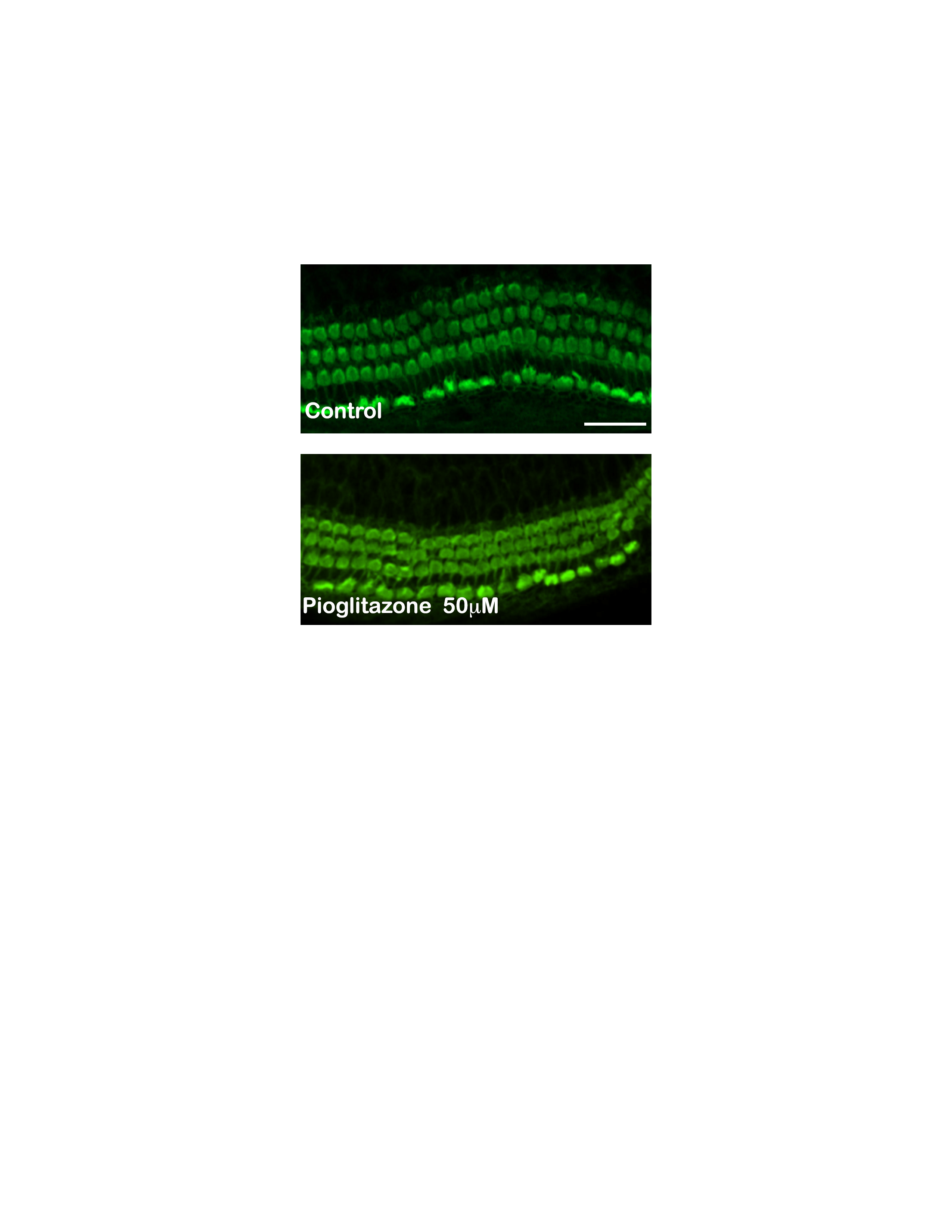

Supplement: S4 Fig — (TIF) [file pone.0188596.s004.tif]

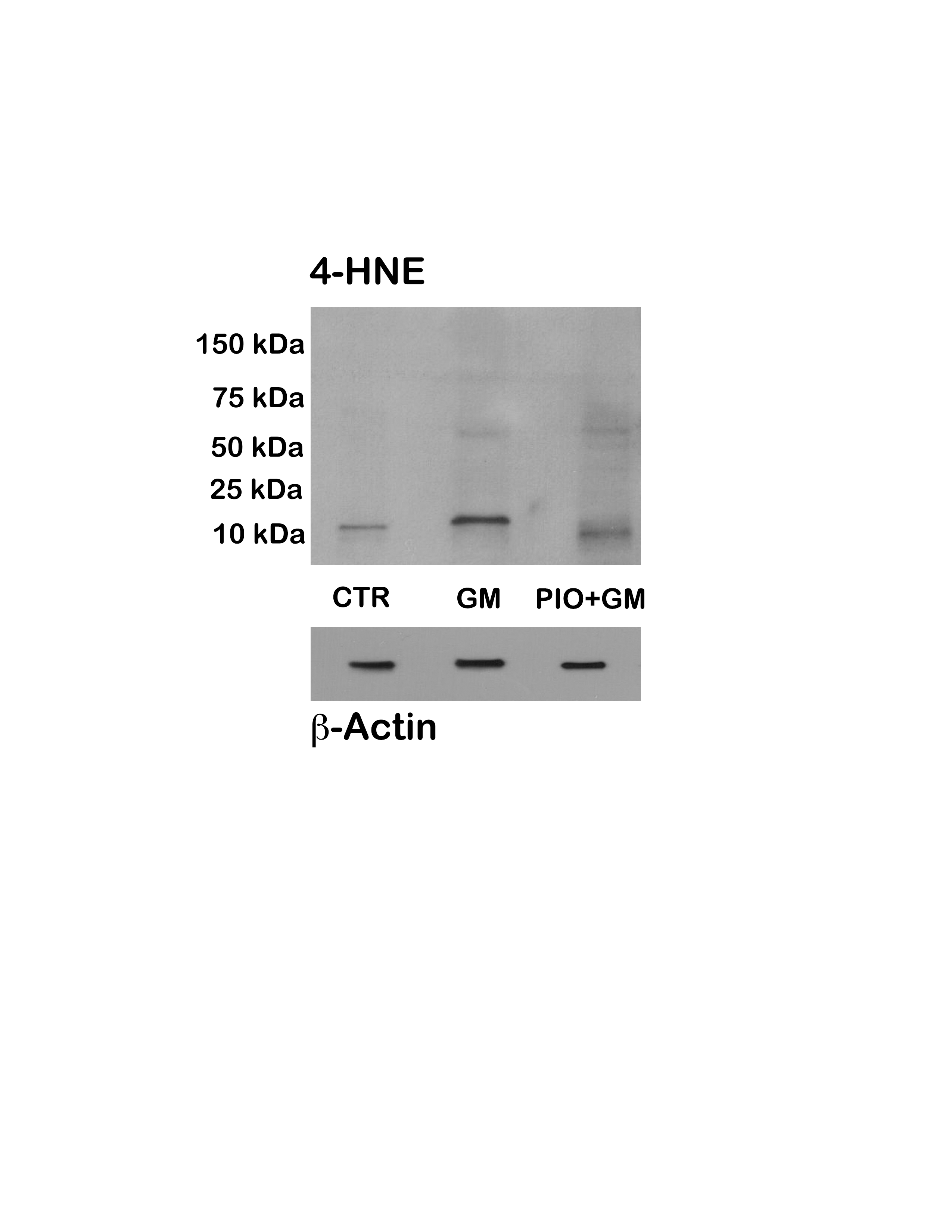

Supplement: S5 Fig — Western blot shows 4-hydroxy-2-nonenal (4-NHE)-modified proteins extracted from mouse organ of Corti (OC). Explanted OCs were untreated (CTR) or exposed to gentamicin (GM), either alone or with pioglitazone (PIO+GM). The blot was probed with the 4-HNE-specific antibody. The results indicate that gentamicin increased 4-HNE modifications, and the addition of pioglitazone prevented 4-HNE damage induced by gentamicin. ß-actin was used as a loading control. (TIF) [file pone.0188596.s005.tif]

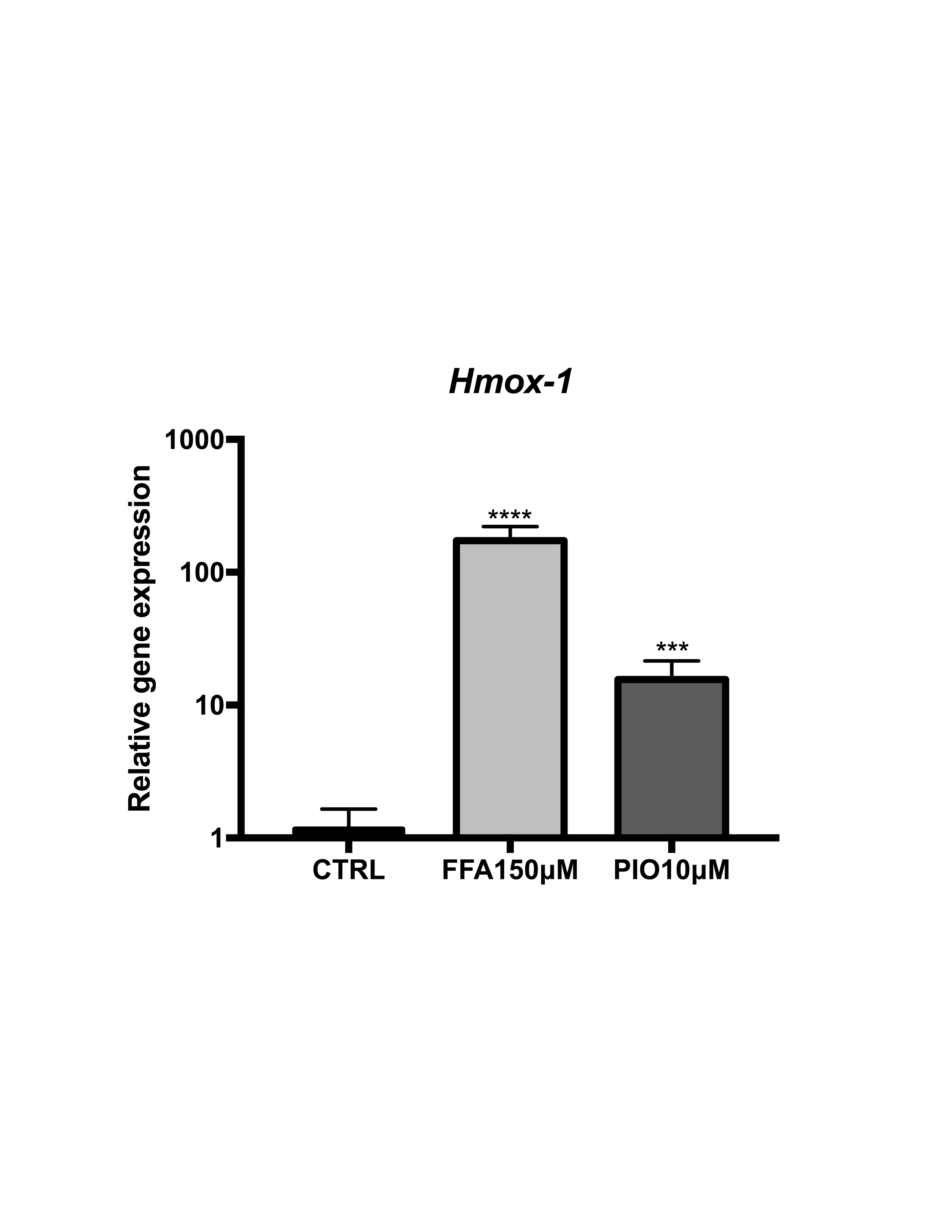

Supplement: S6 Fig — (TIF) [file pone.0188596.s006.tif]
